# Increased triacylglycerol production in *Rhodococcus opacus* by overexpressing transcriptional regulators

**DOI:** 10.1186/s13068-024-02523-3

**Published:** 2024-06-19

**Authors:** Winston E. Anthony, Weitao Geng, Jinjin Diao, Rhiannon R. Carr, Bin Wang, Jie Ning, Tae Seok Moon, Gautam Dantas, Fuzhong Zhang

**Affiliations:** 1 The Edison Family Center for Genome Sciences and Systems Biology, Washington University School of Medicine, St. Louis, MO 63110 USA; 2 Department of Pathology and Immunology, Division of Laboratory and Genomic Medicine, Washington University School of Medicine, St. Louis, MO 63110 USA; 3 Department of Energy, Environmental and Chemical Engineering, Washington University in St. Louis, St. Louis, MO 63130 USA; 4 Division of Biology and Biomedical Sciences, Washington University in St. Louis, St. Louis, MO 63130 USA; 5 Department of Molecular Microbiology, Washington University School of Medicine in St Louis, St Louis, MO 63110 USA; 6 Department of Pediatrics, Washington University School of Medicine in St Louis, St Louis, MO 63110 USA; 7 Department of Biomedical Engineering, Washington University in St. Louis, St. Louis, MO 63130 USA; 8 Institute of Materials Science & Engineering, Washington University in St Louis, St Louis, MO 63130 USA; 9 Present Address: Earth and Biological Systems Directorate, Pacific Northwest National Laboratory, Seattle, USA

**Keywords:** Lignin valorization, Triacylglycerol, Bioproduction, Nitrogen limitation, Phenylalanine metabolism

## Abstract

Lignocellulosic biomass is currently underutilized, but it offers promise as a resource for the generation of commercial end-products, such as biofuels, detergents, and other oleochemicals. *Rhodococcus opacus* PD630 is an oleaginous, Gram-positive bacterium with an exceptional ability to utilize recalcitrant aromatic lignin breakdown products to produce lipid molecules such as triacylglycerols (TAGs), which are an important biofuel precursor. Lipid carbon storage molecules accumulate only under growth-limiting low nitrogen conditions, representing a significant challenge toward using bacterial biorefineries for fuel precursor production. In this work, we screened overexpression of 27 native transcriptional regulators for their abilities to improve lipid accumulation under nitrogen-rich conditions, resulting in three strains that accumulate increased lipids, unconstrained by nitrogen availability when grown in phenol or glucose. Transcriptomic analyses revealed that the best strain (#13) enhanced FA production via activation of the β-ketoadipate pathway. Gene deletion experiments confirm that lipid accumulation in nitrogen-replete conditions requires reprogramming of phenylalanine metabolism. By generating mutants decoupling carbon storage from low nitrogen environments, we move closer toward optimizing *R. opacus* for efficient bioproduction on lignocellulosic biomass.

**Supplementary Information:**

The online version contains supplementary material available at 10.1186/s13068-024-02523-3.

## Introduction

Utilization of biomass, especially lignocellulosic biomass, has enormous unmet potential. Biofuels and commodity chemicals are commonly produced from the cellulose and hemicellulose fractions, but the lignin fraction is frequently treated as a waste stream and burned [1]. Lignin offers promise as a feedstock for microbial production of biofuels and platform chemicals [2, 3]. It has a high energy density with a carbon-to-oxygen ratio greater than cellulosic feedstock [4, 5]. Yet, achieving commercially viable bioproduction is challenging, as lignin is a complex, heterogeneous polymer, and the depolymerization process primarily yields aromatic compounds toxic to many microbes [6, 7].

Unlike most prokaryotes, *Rhodococcus* species have the capacity to produce triacylglycerols (TAGs) as a storage compound, rather than common polyhydroxyalkanoates (PHAs) [8–10]. *Rhodococcus opacus* PD630 (hereafter *R. opacus*) can accumulate up to 80% of its dry cell weight (DCW) as TAGs when grown on gluconate [10], and as much as 44% of DCW when grown on phenol [11], a model compound for lignin breakdown products [11, 12]. TAGs and their derivatives are precursors to aviation fuels and numerous industrial chemicals, representing an area of bioproduction where finely tuned microbial cell factories could make a significant impact [13].

Lipid production in oleaginous *Rhodococcus* species is largely linked to nitrogen stress; in nitrogen-poor growth conditions, cells store carbon in the form of TAGs [14, 15]. When nutrient availability is reversed and carbon becomes scarce in the immediate environment, these storage molecules can then be readily mobilized. Linking lipid production to nitrogen starvation necessarily decouples it from growth, with most TAG accumulation corresponding to stationary phase [16]. Nitrogen limitation presents a challenge for bioproduction on recalcitrant biomass; lipid titers on aromatic substrates can be an order of magnitude lower than on glucose due to additive stress further impairing growth [16]. In *Rhodococcus*, most strategies to improve TAG production have focused on overexpressing native genes, including (1) increasing fatty acid synthesis with the *fasI* operon; (2) boosting the final step in TAG biosynthesis with *atf2*; (3) using thioesterases to increase fatty acid-CoA production; and (4) increasing the NAD(P)H pools via *tadD* or malic enzymes [14, 17–20]. While these methods have improved lipid accumulation in oleaginous *Rhodococcus*, none have addressed the inherent regulation of TAG production under nitrogen limitation or have generated engineered strains designed for utilizing toxic feedstock such as lignin or its breakdown products.

Here, using comparative transcriptomic datasets of *R. opacus* grown under nitrogen and phenolic stress, we identified transcriptional regulators (TRs) predicted to increase lipid titers in a nitrogen-independent manner [12, 21]. We then constructed and tested 27 strains expressing these TRs (see Table S1), three of which demonstrated increased TAG accumulation in nitrogen-replete conditions. Transcriptomic analysis during growth on phenol as a sole carbon source identified a novel expression state during increased lipid production defined by significantly increased expression of phenolic catabolism genes. This novel state, shared by two of the three mutants, modulates expression at multiple levels of phenylalanine metabolism, while simultaneously driving increased expression in cofactor and amino acid metabolism. We further confirmed that overexpressing the phenylacetic acid (*paa*) degradation regulator *paaX* promoted lipid accumulation in nitrogen-replete conditions. Induction of the gene controlling degradation of phenylethylamine (*pea*) and repression of the *paa* degradation operon reveal a complex regulatory mechanism resulting in increased lipid accumulation in nitrogen-rich environments. When grown in nitrogen-replete conditions, Strain 13 successfully increases lipid production with no penalty to growth rate, confirming a decoupling of carbon storage and nitrogen limitation. In summary, we demonstrate that metabolic tuning of phenylalanine metabolism in *R. opacus* leads to increased lipid accumulation. This work represents a critical step toward optimizing *R. opacus* as a chassis for bioproduction.

## Methods

### Chemicals and strains

Unless otherwise indicated, all chemicals were purchased from Sigma Aldrich (St. Louis, MO). The ancestral, or wild-type (WT), strain for all transformant cells was *Rhodococcus opacus* PD630 (DSMZ 44193). This strain was used as a basis of comparison for all engineered strains. Culturing conditions for all experiments, unless otherwise noted, were at 30 °C and 250 rpm, with the previously described minimal salts medium B constituting the growth medium [50]. Media were sterilized using a 0.22-µm filter, with carbon sources added from filter-sterilized stock solutions; nitrogen was added either after pre-sterilization or from a separate filter-sterilized stock solution. Medium pH was adjusted to 7.2 using 6 N HCl or 2 M NaOH solutions. Optical density at 600 nm (OD_600_) was measured using a Tecan Infinite 200Pro plate reader, either directly using VWR semi-micro polystyrene cuvettes or indirectly based on the absorbance at 600 nm (A_600_) measured in black 96-well plates (Greiner Bio-One flat bottom, chimney well, µclear). An A_600_ value can be converted into an OD_600_ value (for *R. opacus* cultures) via the experimentally determined relationship ${OD}_{600}=1.975\times \left({A}_{600}-0.04\right)$. All strains were maintained on tryptic soy broth (TSB) plates supplemented with 1.5% agar. Kanamycin (20 μg/mL), gentamicin (10 μg/mL), chloramphenicol (34 μg/mL), or hygromycin B (200 μg/mL) was added as appropriate to *E. coli* cultures. Kanamycin (50 μg/mL), gentamicin (10 μg/mL), chloramphenicol (15 μg/mL), and/or hygromycin B (50 μg/mL) were added as appropriate to *R. opacus* cultures.

### Plasmid construction and DNA manipulation

All plasmids constructed for this study were confirmed by DNA sequencing (Genewiz; South Plainfield, NJ); all primers were purchased from Integrated DNA Technologies (IDT; Coralville, IA). All overexpression plasmids were assembled using Golden Gate Assembly, and knockout plasmids were assembled using Gibson Assembly. All plasmids were replicated in *E. coli* DH10B, and then isolated using a PureLink™ HiPure Plasmid Miniprep Kit (Invitrogen by ThermoFisher; Waltham, MA) [51, 52]. DNA fragments were amplified using Phusion High-Fidelity DNA Polymerase (NEB; Ipswich, MA) and purified using a ZymoClean Gel DNA Recovery Kit (Irvine, CA). Genomic DNA was extracted from *R. opacus* using a Promega Wizard^™^ Genomic DNA Purification Kit (Madison, WI).

### Transformation of *R. opacus*

Preparation of competent cells was conducted as previously described [50]. In brief, an overnight culture in TSB medium was used to inoculate 100 mL of fresh TSB containing 8.5 g/L glycine and 10 g/L sucrose (initial optical density, OD_600_, diluted to 0.075). Cells were cultivated in standard conditions to an OD_600_ value of 0.4–0.5, corresponding to exponential phase growth. Cells were rapidly chilled and centrifuged at 3.5 k relative centrifugal force (abbreviated as RCF), and then washed twice with chilled, sterile, deionized water. A final resuspension to an OD_600_ ~ 10–15 was made in chilled 10% (v/v) glycerol, and cells were aliquoted at 100 µL and frozen at − 80 °C for later use.

For transformation with a replicating plasmid, approximately 500 ng plasmid DNA was added to prepared electrocompetent cells. The electrocompetent cells were shocked at 2500 mV across a 0.2-cm-gap cuvette (time constant ~ 5–6 ms) and provided with 1 mL rich media (either TSB or SOC/super-optimal broth with catabolites) to recover. For outgrowth, cells were transferred to 50-mL glass culture tubes and incubated under standard growth conditions for 4 h before being spread on TSB plates infused with the appropriate antibiotics. Plated cells were grown at 30 °C for 2–3 days until colonies emerged, and then propagated on fresh plates.

Generation of *R. opacus* knockout mutants was accomplished using a previously developed method for homologous recombination, with modifications [36]. Briefly, electrocompetent cells were prepared as above using a strain expressing a helper plasmid containing the Che9c viral recombinases. Competent cell aliquots were transformed with 1–2 µg of a suicide vector containing the knockout construct (an antibiotic resistance cassette flanked by ~ 500 bp segments homologous to the target gene); the outgrowth period for these transformations was at least 6 h and up to 12 h. Transformed cells were plated on TSB with the corresponding antibiotics and incubated at 30 °C for 4–5 days; colonies were propagated on fresh TSB plates and verified by colony PCR (Promega GoTaq^®^ G2 DNA Polymerase).

### Fermentation for lipid analysis

Frozen stocks of strains (generated from isolated colonies and stored at − 80 °C) were streaked onto fresh TSB plates, with antibiotics as appropriate, and then grown for 2–4 days. A loopful of cells were used to inoculate seed cultures in minimal media B with 1 g/L each (NH_4_)_2_SO_4_ and glucose as nitrogen and carbon sources, respectively [50]. These cultures were centrifuged at 3.5 k RCF, and the pellets were resuspended in low-nitrogen minimal media. OD_600_ of these cell suspensions was adjusted to approximately 2, and then used to inoculate the 50 mL fermentation cultures (250-mL non-baffled Erlenmeyer flasks). The carbon conditions were either 2 g/L glucose or 0.4 g/L phenol, and the nitrogen conditions were either 0.05 g/L or 1 g/L (NH_4_)_2_SO_4_ (hereafter ‘low’ and ‘high’ nitrogen), for a total of four combinatorial conditions. Each strain was grown in one flask per carbon/nitrogen condition. Cultures were grown in standard conditions for 72 h unless otherwise described. Final OD_600_ was measured and used to calculate the volume necessary to collect 5 OD units of cells (or 10 OD units, in the case of the glucose/low nitrogen condition) using the relation $Vol=5/{OD}_{600}$. Triplicate samples of each strain and culture condition were collected and centrifuged at 3.5 k RCF for 10 min, and then the culture supernatant was discarded, and the cell pellets were stored at − 20 °C prior to lipid extraction. The low nitrogen concentration (0.05 g/L (NH_4_)_2_SO_4_) was chosen to optimize lipid titer in phenol cultures, and the high-nitrogen condition (1.0 g/L (NH_4_)_2_SO_4_) is sufficient for nitrogen-replete growth and lipid accumulation in both carbon conditions.

### Lipid extraction and analysis

An acid–chloroform lipid extraction was performed as described [22], with modifications. In brief, the pelleted 5 (or 10) OD units of cells were resuspended in 100 µL sterile, deionized H_2_O and transferred to a 15 mL glass centrifuge tube. 1 mL of 10% (v/v) H_2_SO_4_ in methanol was added to the cells, as well as 1 mL of chloroform and a C_12_ standard (40 mg/mL lauric acid dissolved in methanol) to a final concentration of 40 mg/L. Cell solutions were incubated at 100 °C for one hour, and then chilled rapidly on ice prior to adding 1 mL of deionized H_2_O and mixing thoroughly by vortex. Finally, the cell extracts were centrifuged at 1 k rpm for 5 min at room temperature, and the resulting organic layer was extracted into GC vials [49]. Samples were stored at 4 °C prior to analysis.

Lipid extracts were analyzed as previously described via GC-FID using an Agilent 6890A system equipped with a DB5-MS column and a flame ionized detector (FID) (Agilent Technologies) [23, 24]. A 1 µL sample of each lipid extract was injected into the 250 °C inlet, with N_2_ carrier gas flowing at 1.4 mL/min. Each sample run began with an initial oven temperature of 80 °C, and then ramped at 20 °C/min to 300 °C, where it held for a final three minutes. Between samples, the injector was washed with ethyl acetate, with ethyl acetate also serving as the blank samples at the start and end of each sample run. Peak integration was carried out using the ChemStation software and exported to Microsoft Excel for data processing. In addition to the C_12_ internal standard, the components of the lipid samples were quantified compared to standard curves of 13 fatty acids, detailed in Table 2S [25–27]. All standard curves were plotted by the area of the peaks of 25 mg/L, 50 mg/L, and 100 mg/L standard compounds. The fitted slopes of these curves were used to calculate the concentration of each component within the extracted lipid samples matched by retention time. 40 mg/L of lauric acid (C12) was added as internal standard for the normalization of different samples. If necessary, summed average titers were transformed to standardize control samples between different fermentation experiments.

### RNA extraction and rRNA depletion

RNA extraction proceeded as previously described [11]. Briefly, RNA was extracted using the RNA MiniPrep kit (Zymo Research) and treated with two doses of TURBO DNase I (Ambion) for 30 min at 37 °C to remove DNA contamination. DNase-treated RNA was then cleaned using the RNA Clean & Concentrator Kit (Zymo Research), and then tested for DNA contamination by PCR amplification using intergenic primers. Any samples with distinct bands after PCR were digested and cleaned again until no DNA was detected. Total RNA concentration was quantified using a NanoVue Plus spectrophotometer, and rRNA was depleted using the Bacterial Ribo-Zero rRNA removal kit (Illumina). mRNA was converted to cDNA and barcoded using previously described methods [12]. cDNA samples were then pooled in equimolar ratios and diluted in nuclease-free water to a final concentration of 10 nM for sequencing.

### Sequencing library preparation and transcriptomic analysis

A 20 uL equimolar mix of cDNA samples were submitted for sequencing at DNA Sequencing Innovation Lab (DSIL) in the Edison Family Center for Genome Sciences and Systems Biology at Washington University in St. Louis School of Medicine. Samples were single-end sequenced (1 × 75 bp) using the Illumina Hi-Seq 2500 System.

**Fig. 1:**
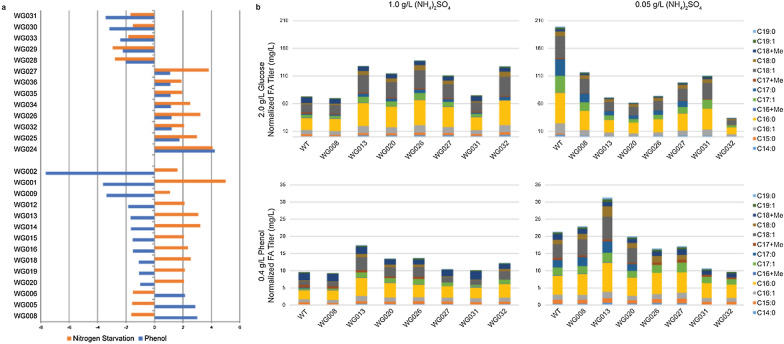
Overexpressing endogenous transcription factors (TFs) that show strong differential expression when grown in phenol and with nitrogen starvation results in increased TAG production in nitrogen-rich environments, compared to the wild-type strain. **a** Using two previously published datasets [12, 36], we identified TFs which were differentially expressed in phenol and during nitrogen starvation, compared to nutrient broth. The 27 genes which had greater than twofold change were selected for overexpression. **b** A subset of the 27 successfully overexpressed TFs were selected for FA production. 7 Strains were grown in either glucose or phenol at two different nitrogen concentrations—representing nitrogen-rich and nitrogen-poor environments—for 60 h, and then lipids were extracted and measured using GC-FID. pWG013- and pWG026-containing cells produced higher lipid titers than WT in three conditions and were chosen along with the pWG020-containing strain for transcriptomic analysis. All lipid titers have been quantified using an internal C_12_ standard at 40 mg/L and averaged across technical replicates

After demultiplexing, raw reads were trimmed using trimmomatic [28] with the standard settings and the CROP length of 75 bp. Samples with more than 15 million trimmed reads were subsampled using seqtk [29], and then mapped to a bowtie2 library built based on the ASM2054278v1 *R. opacus* reference (Refseq assembly GCF_020542785.1), sorted, and indexed. Expression counts were calculated for each genetic loci using featureCounts [30] and imported into R [31] for statistical analysis and visualization. Differential gene expression analysis was performed using DESeq2 [32], and pathway enrichment was performed using gage with the following parameters: test = ”unpaired”, set.size = c(10,100), same.dir = TRUE, rank.test = FALSE, and test4up = TRUE. Counts were transformed using the regularized log transformation in DESeq2 [32]. Heatmaps were generated in R using pheatmap [33]. An initial assessment of expression coverage across the genome identified a lack of expression from all genes located on plasmid 3 in WT grown in both phenol and glucose, as well as a majority of genes located on plasmid 2 in Strains 13 and 20 grown with both carbon sources (Fig. 1S). We thus removed those plasmids from analysis and concentrated our assessment of differential expression (DE) on loci present in all conditions. The 500 most highly variable transcripts were identified from transformed data. The chord distance metric was calculated using the disttransform function from BiodiversityR [34] for generating ordination analysis. Redundancy analysis (RDA), beta dispersion, and PERMANOVA were conducted using vegan [35] in R. Genes associated with altered transcriptomic states were identified by selecting the top 100 loading vectors with directions closest to the centroid of the Strain 13 samples. Any genes that were not annotated to a symbol name were omitted. The trimmed, subsampled cDNA sequences were uploaded as fastq files to the Sequence Read Archive (https://www.ncbi.nlm.nih.gov/sra) in bioproject PRJNA1039565.

## Results

To identify TRs required for nitrogen-independent lipid production in *R. opacus*, we utilized two previously published transcriptomic datasets [12, 36]. Specifically, we focused on TRs that were differentially expressed (DE) under both nitrogen and phenolic stress. Of the 399 TRs genes considered, 141 responded with at least a twofold change in transcription when grown in phenol, and 216 responded to low nitrogen. This pool was narrowed to 33 candidates which responded with > twofold changes to both stimuli (Fig. 1a). 8/33 (30%) TRs, including K2Z90_RS35615 (WG027), K2Z90_RS02690 (WG026), and K2Z90_RS00365 (WG035), were overexpressed under both stress conditions (Fig. 1a). And 11/33 (41%) TRs, including K2Z90_RS32760 (WG013) and K2Z90_RS14485 (WG020) decreased expression in phenol and increased in nitrogen starvation. The observed changes in the expression of 33 TR under diverse lipid production conditions highlight the potential for controlling TAG production through engineering.

### Overexpression of *R. opacus* transcriptional regulators increases lipid accumulation when grown on glucose or phenol

These 33 TRs were cloned into the high-copy pAL5000(S) plasmid under a strong constitutive promoter; 27 were successfully overexpressed in *R. opacus* (see Supplementary Table 1 for a full list). We then used the lipid-staining dye NileRed to screen for their lipid accumulation under both phenol and nitrogen starvation stress. From the list of 27 TRs, we selected 7 (Fig. 1b): strains WG027 (1), WG026 (2), and WG035 (3) overexpress TRs K2Z90_RS35615, K2Z90_RS02690, and K2Z90_RS00365, strain WG031 (4), strain WG008 (5) and strain WG013 (6) overexpress TR K2Z90_RS09960, K2Z90_RS10170 and K2Z90_RS32760, respectively, and finally strain WG020 (7) expresses K2Z90_RS14485.

These 7 strains were tested in fermentation using either glucose or phenol as a sole carbon source with or without nitrogen limitation (Fig. 1b). In comparison to wild-type (WT) expressing an empty vector control, five of seven characterized mutant strains produced higher titers of lipids (FAs) in at least one of the four fermentation conditions: 2.0 g/L glucose with either 1.0 g/L or 0.05 g/L (NH_4_)_2_SO_4_, and 0.4 g/L phenol with the same concentrations of (NH_4_)_2_SO_4_ (Fig. 1b). Importantly, strain WG013 (hereafter called Strain 13) and WG026 (hereafter called Strain 26) increased FA production in both nitrogen-rich and poor conditions in phenol, highlighting their potential for converting lignin into TAG. Additionally, strain WG020 (hereafter called Strain 20) produced higher FA titers than WT in the presence of high (NH_4_)_2_SO_4_ under both the glucose and phenol carbon source. Based on these measurements, we selected these three strains (Strains 13, 20, and 26) alongside WT for further transcriptomic analysis to understand the regulatory roles of these TRs.

### Strains 13 and 20 induce transcriptional reprogramming in phenol and glucose

**Fig. 2:**
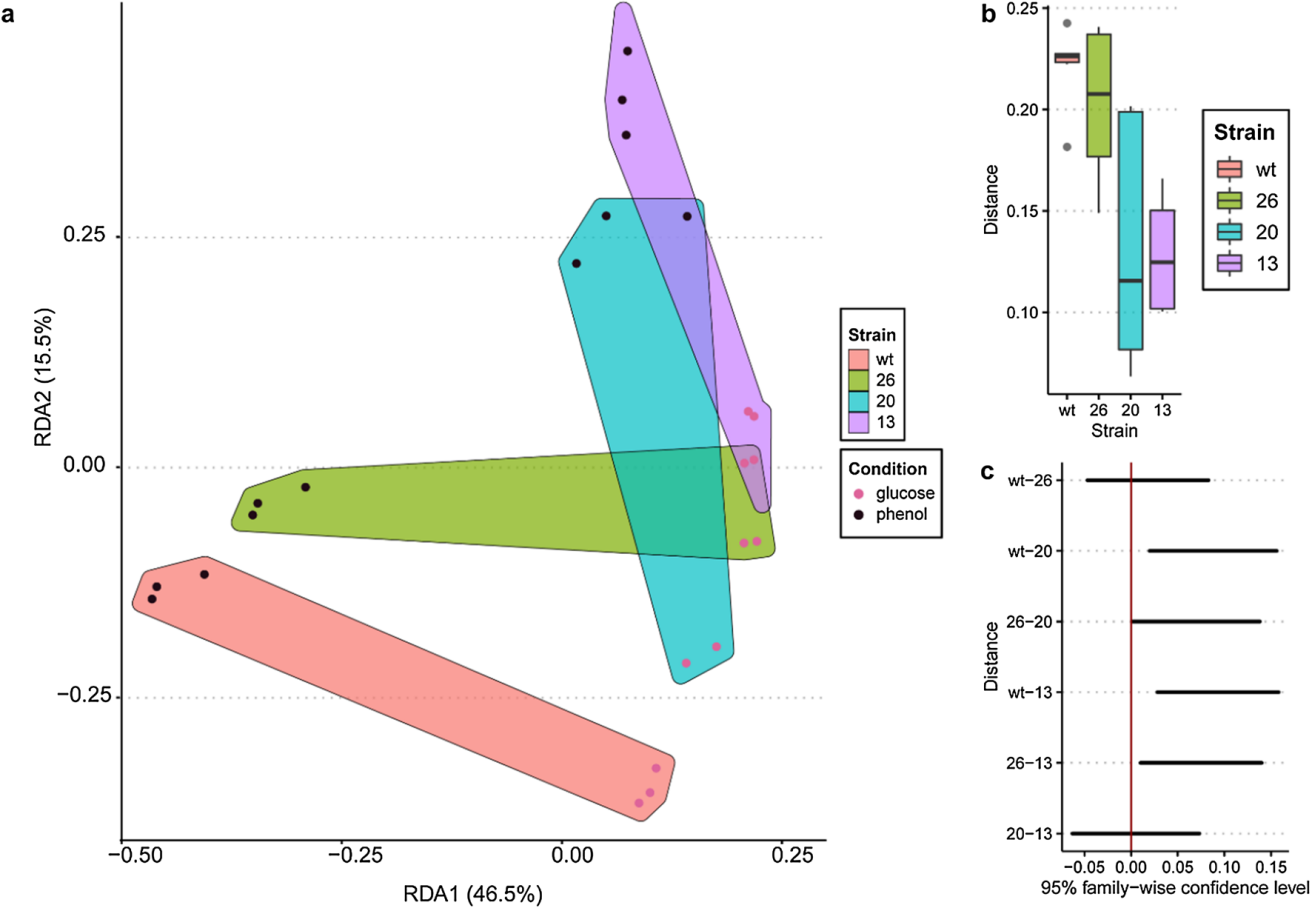
TR expression induces transcriptional reprogramming across strains and carbon sources. **a** Redundancy analysis (RDA) was conducted on the first two principal component axes generated from the 500 most highly variable transcripts across all strains. Samples from each strain are illustrated by colored hulls. **b** Distance to centroid boxplots for each strain. The centroid of all the samples from a strain (across both conditions) was generated. **c** 95% family-wise confidence level of the difference in dispersion between strains. A vertical line demarcates zero; a confidence interval which crosses this line indicates the true difference between the dispersion of the two conditions is likely zero

Using constrained ordination (RDA), we found that strain identity and carbon source significantly associated with RNAseq profiles of each sample (redundancy analysis for strain and carbon source, p = 0.0019 and 0.0005, respectively; permuted 2000 times; Fig. 2a). We detected large dispersion of the transcriptome samples grown in phenol, and we confirmed a significantly lower distance to centroid for all samples grown in glucose (permutation test for homogeneity of multi-variate dispersions, p = 0.001, 999 permutations). Beta dispersion analysis identified significant differences between Strains 13 and 20 compared to both WT and Strain 26 (permutation test for homogeneity of multi-variate dispersions, p = 0.002, 999 permutations, [Fig. 2b] followed by Tukey’s HSD post hoc test of the permuted dispersions [Fig. 2c]). The transcriptional profiles of Strains 13 and 20 were compositionally similar (permuted analysis of variation, PERMANOVA, p = 0.169), indicating that overexpression of TR K2Z90_RS32760 and K2Z90_RS14485, while significantly affecting global expression, produces similar effects on the cell. Interestingly, the expression profile of Strain 26 exhibits composition close to that of WT (Tukey’s HSD, p = 0.86; and PERMANOVA, p = 0.267,), while still increasing TAG production.

### Strains 13 and 26 grown in phenol increase expression of translation-associated and phenol utilization genes

**Fig. 3:**
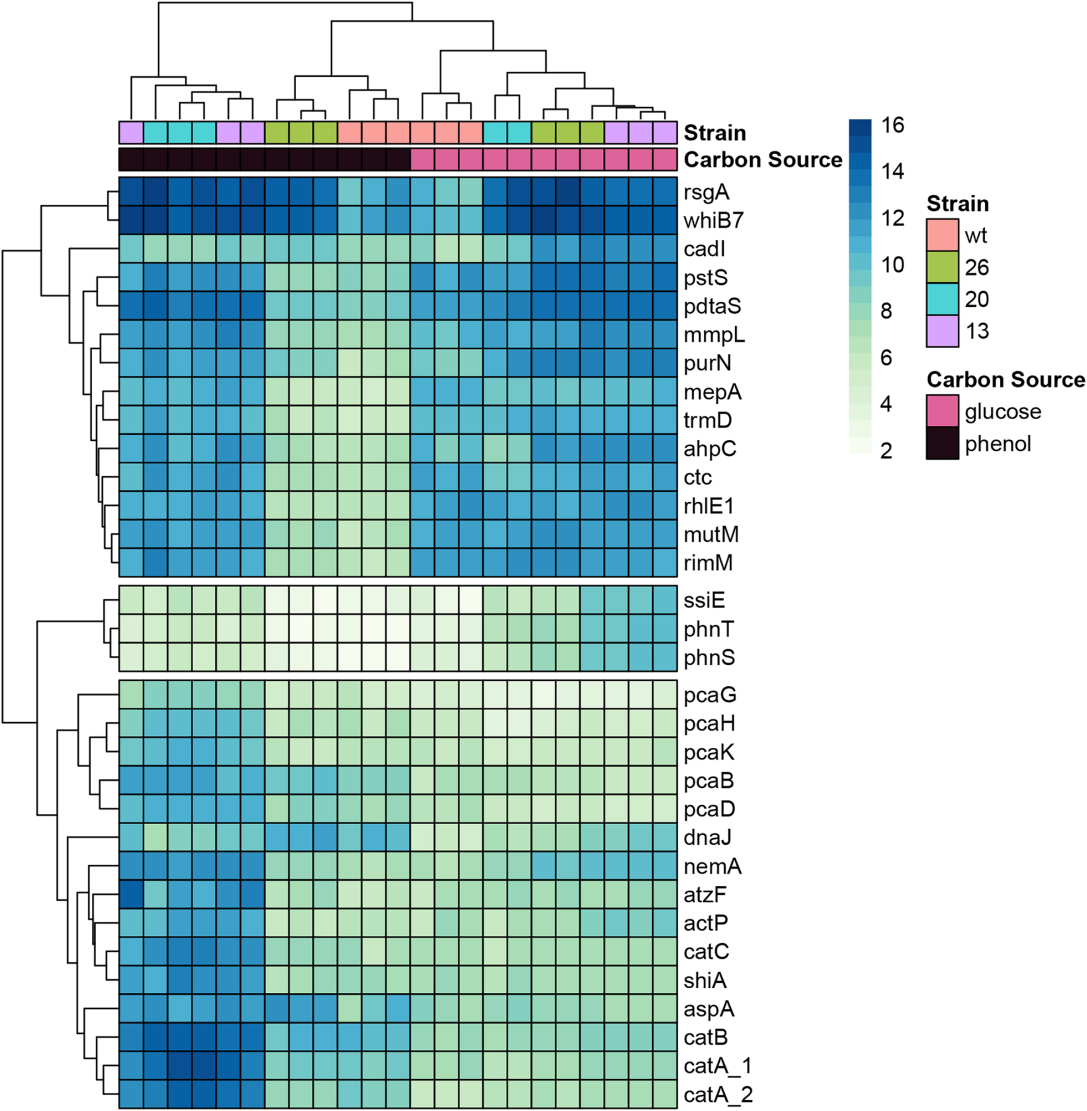
TR 13 and 20 induce activation of carbon-specific alternate transcriptional programs. A heatmap of the loci with RDA loadings lying closest to Strains 13 and 20. Heatmap values are regularized-log transformed expression counts. Column annotations indicate the strain identity and carbon source. Rows are clustered into three groups: (1) genes upregulated in glucose in WT that become universally upregulated in Strains 13 and 20 in either glucose or phenol; (2) a set of three genes which are only upregulated in Strain 13 grown in phenol; and (3) genes upregulated in Strains 13 and 20, compared to WT

We next identified the genetic components significantly associated with the altered transcriptomic state seen in Strain 13, which is the strain with the most significant difference in homogeneity and composition from WT (Tukey’s HSD, p = 0.0035, Fig. 2b and c, and PERMANOVA, p = 0.0032). These clustered into three distinct groups based on expression: (1) a set of genes with low expression in WT and Strain 26 grown in phenol but highly expressed in Strain 13 and Strain 20 regardless of carbon source (hereafter referred to as “carbon source universal optimization gene set for TAG production” or COG), (2) a set of three genes (the transport genes *phnT* and *phnS* and the biofilm promoting virulence factor gene *sslE *[37]) that were only upregulated in Strain 13 grown in glucose, and (3) a set of genes differentially expressed in Strains 13 and 20 versus WT grown in phenol (hereafter referred to as “phenol-specific optimization gene set for TAG production” or POG) (Fig. 3). COG contains a set of genes involved in translation (*rhlE1, cpc, rimM,* and *trmD *[38, 39]) and *pstS* (encoding phosphate transport) [40]. A subset of the third set (*rsgA, purN, phnT/S, pdtaS, pstS,* and *ahpC*) was significantly upregulated in Strain 13 versus WT, when grown on glucose.

The POG contained a set of genes highly upregulated in Strains 13 and 20 compared to WT. This included the *catABC* gene cluster of the ortho cleavage arm of catechol degradation [41], the *pcaBDHGK* genes of the protocatechuate degradation II pathway (both of which are important for degradation of aromatic carbon sources) [42], and the shikimate transporter *shiA *[43].

### Strain 13 induces upregulation of metabolic pathways related to protein and cofactor synthesis in phenol

**Fig. 4:**
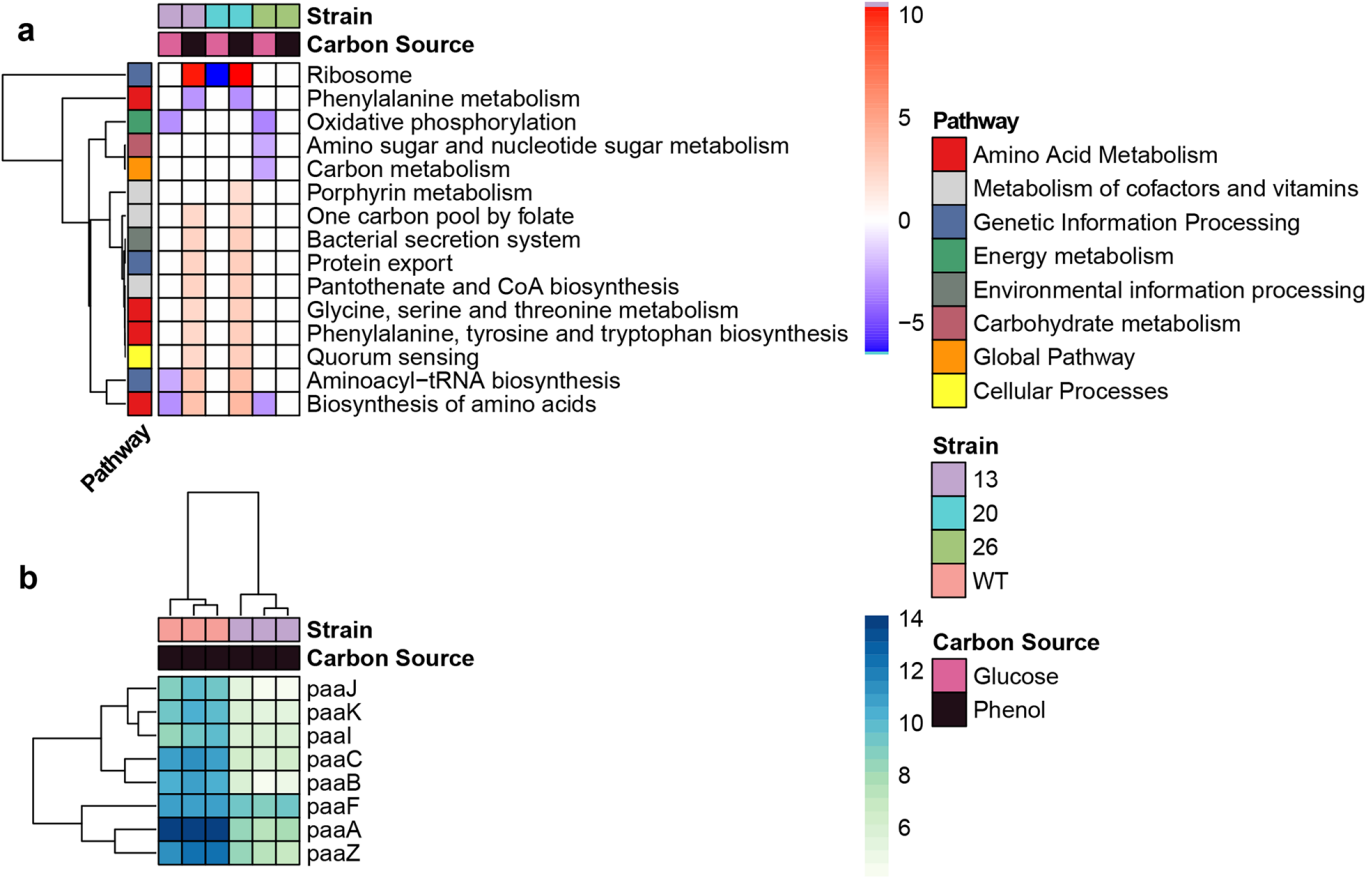
The PaaX-like TR 13 induces carbon-specific alternate transcriptional programs and represses phenylacetic acid degradation. **a** Heatmap visualizing the differential expression of KEGG pathways. Each mutant was compared to the WT strain grown using the same carbon source for differential expression in all the *R. opacus*’ annotated KEGG pathways (rows). The color of each cell of the heatmap denotes the fold change versus WT, and white cells represent non-significant changes. Each column represents the DE of one condition, averaged from replicates and tested using GAGE in R. Annotation bars on top of the heatmap denote the strain and carbon source in each column, and the row annotation bar on the side denotes the BRITE functional hierarchy classification for each pathway. Rows are clustered using the “complete” distance method. **b** Heatmap visualizing the DE of the phenylacetic acid degradation pathway in Strain 13 versus WT grown on phenol. Here, all loci annotated to the phenylacetic acid pathway were shown as rows, and each column represents one of the replicates for each condition. Column annotation bars on top of the metric indicate the strain and carbon source designation of each replicate. Rows and columns are clustered using Euclidean distance and the “complete” clustering metric. Heatmap values are regularized-log transformed expression counts

Strain 13 overexpresses K2Z90_RS32760, which is annotated as a PaaX-family TR. We conducted KEGG pathway analysis to determine which cellular components underwent differential expression, comparing all strains to WT in both carbon sources (Fig. 4a). TR 13 downregulated three KEGG pathways when grown in glucose: oxidative phosphorylation, aminoacyl-tRNA biosynthesis, and the biosynthesis of amino acids, while TR 20 only downregulated the KEGG ribosome pathway. Strain 13 grown in phenol increased expression of ten KEGG pathways, including pantothenate and CoA metabolism, phenylalanine, tyrosine, and tryptophan biosynthesis, and ribosome and aminoacyl-tRNA synthesis. TR 20 upregulated the same set of ten pathways, but also upregulated porphyrin metabolism. Both TR 13 and TR 20 upregulated multiple metabolic and cofactor pathways as well as pathways related to amino acid metabolism. Strain 26 did not upregulate any pathways in either carbon condition, and instead downregulated four categories in glucose: oxidative phosphorylation, amino and nucleotide sugar biosynthesis, carbon metabolism, and biosynthesis of amino acids. Strain 26 did not differentially express any pathways versus WT in phenol.

To confirm TR 13’s role in regulating the *paa* pathway, we examined differential expression at the loci annotated to be a part of the *paa* cluster (Fig. 4b). All genes within the cluster were significantly downregulated in Strain 13 grown in phenol compared to WT. Differential expression of KEGG modules within the phenylalanine, tyrosine, and tryptophan metabolism and phenylalanine degradation pathways further identified the “phenylacetate degradation, phenylacetate to acetyl-CoA/succinyl-CoA” module as being significantly downregulated in all mutant strains grown in phenol (Figure S1, see Fig. 4S for KEGG reference pathway). All three strains also exhibited upregulation of the “tryptophan biosynthesis, chorismate to tryptophan”. Strains 13 and 20, but not 26, also upregulated the “shikimate pathway, phosphoenolpyruvate + erythrose-4P to chorismate” module.

### Strain 13 increases FA titers by remodeling phenylalanine metabolic pathway expression and exhibits WT-like growth in nitrogen-replete glucose conditions

**Fig. 5:**
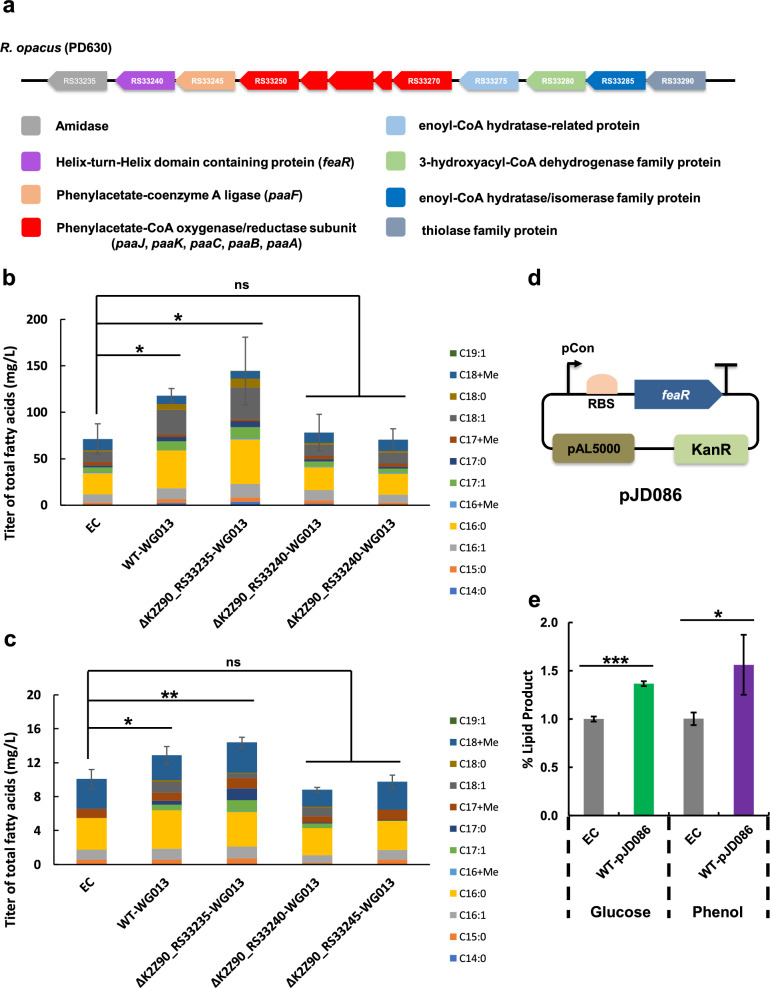
Increasing TAG production through altered regulation of phenylalanine degradation. **a** The proposed phenylacetic acid (PAA) degradation operon. Gene symbols displayed for all annotated loci using reference genome ASM2054278v1. **b**, **c** Assays of lipid profiles of mutant strains with cultivation on 2 g/L glucose (**b**) or 0.4 g/L phenol (**c**) as carbon source. All mutant strains harbor the *paaX* overexpression plasmid pWG013. The WT strain harboring an empty vector was used as a control (EC). **d** Schematic diagram of the replicating plasmid used for overexpressing the *feaR* transcription factor. **e** Lipid production of the pJD086 strain overexpressing *feaR*. The control (EC) and the engineered strains were cultivated on both 2 g/L glucose and 0.4 g/L phenol. All assays were conducted in nitrogen-replete conditions (1 g/L (NH_4_)_2_SO_4_). All values represent the mean from triplicate cultures, with error bars depicting the standard deviation from that mean. Unpaired two-tailed *t*-test was used to compare the variation in the change in lipid contents of the mutants against that of the EC strain (ns, not significant). Asterisks denote statistical significance of comparisons: * : p < 0.05, ** : p < 0.01, *** : p < 0.001

PaaX encoded by K2Z90_RS32760 is a negative regulator acting on an 8-gene phenylacetic acid degradation (*paa*) gene cluster, which is located a few kilobases downstream of *paaX* in the *R. opacus* genome. As shown previously (Fig. 1b), Strain 13 overexpressing K2Z90_RS32760 resulted in a significant increase in FA titer (two-tailed t-test, p = 0.011, standard deviation of 7.76). To determine whether altering the functionality of different components of the *paa* pathway modified the increased FA titer phenotype, an antibiotic selection marker was site-specifically inserted into three consecutive genes within the PAA degradation pathway (from upstream to downstream): K2Z90_RS33245, K2Z90_RS33240, and K2Z90_RS33235 which encode the phenylacetate-coenzyme A ligase (PaaF), a helix-turn-helix transcriptional regulator (FeaR), and an amidase, respectively (Fig. 5a).

While deleting the amidase in the presence of overexpressed K2Z90_RS32760 resulted in a further increase in FA titers (Fig. 5b), FeaR or PaaF deletion resulted in loss of the increased FA titer, returning to WT’s production levels (Fig. 5a and b, glucose and phenol, respectively). Because disruption of PaaF expression via selection marker integration simultaneously disrupts FeaR expression, we conclude that PaaX enhances FA production in *R. opacus* by activating FeaR expression or repressing the *paa* pathway. FeaR is responsible for the activation of the upstream *pea* degradation pathway [36], which produces phenylacetate (Fig. 4S). To further confirm the role of FeaR in regulating FA production in *R. opacus*, we overexpressed *feaR* (Fig. 5d and e) without K2Z90_RS32760 overexpression. Lipid profile analysis revealed that in nitrogen-replete conditions with either glucose or phenol as carbon source, lipid titer was significantly increased by 36.6% (two-tailed t-test, p < 0.0001, standard deviation of 0.025) and 56.2% (two-tailed t-test, p = 0.0375, standard deviation of 0.309), respectively (Fig. 5e). This result suggests that activating the *pea* degradation pathway enhanced FA production in a similar manner to the change resulting from repression of the downstream *paa* pathway (Fig. 4S).

 Finally, to determine whether the increased lipid accumulation in Strain 13 would result in a decrease in biomass as previously described [16], we grew Strain 13 and WT on glucose. We varied the concentration of ammonium sulfate to simulate nitrogen-replete (1.0 g/L (NH_4_)_2_SO_4_, Figure S3a) and nitrogen-limited (0.05 g/L (NH_4_)_2_SO_4_, Fig. 3Sb) conditions. In nitrogen-replete conditions, Strain 13 and WT grew at the same rate, while in nitrogen-limiting conditions, Strain 13 exhibited a decrease in max OD_600_.

## Discussion

Carbon storage in the bacterial cell is usually induced during nutrient limitation (e.g., phosphorus and nitrogen limitation) and often occurs in stationary phase [44–46]. Genetic components involved in modulating TAG synthesis in *R. opacus* PD630 have been discovered [14, 15], but identifying regulatory elements which adjust lipid accumulation in phenol and other recalcitrant feedstocks is important for optimizing *R. opacus* PD630 for efficient lignin valorization. In this study, we increase bacterial lipid production (even during nitrogen-replete conditions) through overexpression of endogenous transcriptional regulators, examine the effects of these modulations on the transcriptome, and use experimental techniques to interrogate their mechanisms further.

Of the seven TR overexpression mutants selected for FA titer measurement, Strains 13, 20, and 26 exhibited higher FA titers (Fig. 1). We observed the creation of two unique transcriptional states. TR 26, which produces a statistically similar level of dispersion and composition to that of WT *R. opacus*, induces a markedly different transcriptional regime than that of TR 13 and 20 and little to no pathway differential expression versus WT. While the mechanism demarcating the transcriptional state shared by Strains 13 and 20 versus 26 is unclear, we show that increasing TAG production in *R. opacus* is possible through multiple unique genetic modifications. Understanding these mechanisms may enable strategies to bypass the requirement of nitrogen starvation for TAG production. Nevertheless, these observed changes in lipid accumulation highlight potentials in controlling TAG production by engineering TRs.

Conversely, overexpressing the nitrate regulatory protein NarL (TR 31) yielded minimal changes to FA titers. NarL plays a role in regulating the activity of terminal electron receptors depending on environmental cues [47] and sensing nitrogen intermediates, and it is associated with biofilm formation and host-associated survival in bacteria [48]. The lack of an effect suggests that the mechanisms for decoupling the relationship between TAG accumulation and nitrogen availability are only tangentially related systems acting directly on nitrogen and its intermediates. This observation is further supported by observing the functions of TR 13, 20, and 26 to be unrelated to nitrogen resource regulation in *R. opacus*.

Given the end goal of increasing TAG production, it was surprising that so few of the classical lipid biosynthesis and metabolism genes were identified as differentially expressed. Several genes associated with increased TAG production have been identified in *Rhodococcus* species. The major lipid droplet protein, TadA [49], required for lipid storage and droplet formation, was significantly upregulated in Strains 13 and 20. To a lesser extent, MLDSR, the regulator which controls *tadA* expression [50] was also upregulated in Strain 26. We also identified differential expression in the orthogonal biochemical KEGG pathway Coenzyme A biosynthesis. Coenzyme A overexpression to increase TAG biosynthesis in oleaginous microbes has already been successfully implemented in algae [51], suggesting that further modification of Coenzyme A metabolism is a promising method for increasing TAG production without direct modulation of the tightly controlled lipid biosynthesis metabolic circuit. More work thoroughly investigating the link between *tadA* expression, nitrogen regulation, and Coenzyme A metabolism is needed to determine the connection between these metabolic components.

TR 13 and 20 overexpression resulted in similar effects on the transcriptome and led to repression of the *paa* gene cluster. This alteration of phenylalanine metabolism was integral toward decoupling carbon storage from nitrogen limitation in these strains, resulting in upregulation of genes within the *cat* and *pca* branches of the β-ketoadipate catabolism pathways and improved utilization and lipid accumulation in a nitrogen-replete phenolic environment at little cost to growth. Though previous work has shown phenol to be utilized solely through the *cat* branch [11], upregulation of both branches of the β-ketoadipate pathway was also seen in WT and in descendant strains of *R. opacus* PD630 adapted on multiple aromatic compounds [11]. The transcriptional state of mutant strains grown in glucose exhibited far less compositional change than mutants grown in phenol. The upregulated COGs were not involved with glucose metabolism, but instead protein translation (*rhlE1, cpc, rimM,* and *trmD*). This is likely due to the increased synthesis demand during protein overexpression. The lack of carbon source-specific gene regulation demonstrates that glucose metabolism is not the limiting step during TAG accumulation in nitrogen-replete environments.

Beyond phenylacetic acid metabolism, we further demonstrate that alteration of upstream modules of the phenylalanine metabolism pathway also leads to altered lipid accumulation. *feaR* encodes for the activator of the *pea* degradation pathway, which produces phenylacetate [52]. Overexpression of *feaR* alone was sufficient to increase TAG production, and a Δ*feaR/* + *paaX* strain resulted in a loss of increased TAG production. The strain with the largest increase in FA titers was Δ*K2Z90_RS33235*, the gene encoding for an amidase located directly adjacent to the *paa* pathway. There is growing evidence that phenylacetate is a cross-kingdom signaling molecule with important contributions to the oxidative stress response in *Acinetobacter baumannii,* and it is known to regulate growth and development in plants [53]. Given that deleting genes near the *paa* pathway increases FA titers and that deleting genes within the *paa* gene cluster resulted in loss of increased FA titers, it seems likely that a functional *paa* is still required within the cell, perhaps to allow *R. opacus* PD630 to efficiently respond to the lipid peroxidation associated with increased oxidative stress [53].

We thus hypothesize that altering the expression of either the *pea* pathway activator or the *paa* pathway repressor, both modules of the phenylalanine metabolism super-pathway, leads to increased expression of the phenol degradation pathway. Simultaneously, altered expression of cofactor resources such as Coenzyme A may beneficially modulate resource availability for the phenol degradation pathway, as has been shown in phenolic acid production in *S. cerevisiae *[54]. This could result in the increased expression of the POG gene set when in the presence of phenol and an increase in lipid production without the growth attenuation traditionally observed in nitrogen-replete conditions. ^13^C-metabolic flux analysis would provide a more complete understanding of how these genetic alterations affect carbon flow through central metabolism and illuminate the true effects of these observed transcriptomic changes. In conclusion, we have demonstrated the ability to increase TAG production through the overexpression of endogenous transcriptional regulators. The effects on the transcriptome were significantly greater when grown in phenol and resulted in a decoupling of the traditional trade-off between growth and carbon storage. This work describes a novel mechanism for improving TAG production in phenol and glucose toward achieving an eventual goal of advancing lignin valorization.

### Supplementary Information

Supplementary Table S1 Overexpression strains screened for nitrogen-independent lipid production in Rhodococcus opacus PD630. Table S2. Fatty acid standards for analysis of lipid samples extracted from Rhodococcus opacus PD60 cells. Figure S1. Potential native plasmid loss in multiple strains on growth in phenol and glucose. Figure S2. KEGG modules within the phenylalanine, tyrosine, and tryptophan biosynthesis and phenylalanine metabolism pathways with significant DE. Heatmap of KEGG module enrichment in each strain and carbon source. Each mutant was compared to WT grown using the same carbon source for differential expression in all the R. opacus’ annotated KEGG modules (rows). The color of each cell in the heatmap denotes the fold change versus WT, with white cells representing non-significant changes. Each column represents the DE of one condition, averaged from replicates and tested using GAGE in R. 33 Annotation bars on top of the heatmap denote the strain and carbon source in each column. Rows are clustered with the “complete” distance method using Minkoswki distance. Figure S4. KEGG reference pathway of phenylalanine metabolism, including the phenylacetic acid and phenylethylamine degradation modules. Network diagram representing gene/genes as rectangles and metabolites as nodes. Outlets to other reference pathways are denoted as ovals.

## Data Availability

No datasets were generated or analyzed during the current study.

## References

[1] Harkin, J.M., *Lignin and its uses*. Forest products lab madison wis. 1969.

[2] Chatterjee A (2020). Bioconversion of renewable feedstocks by *Rhodococcus opacus*. Curr Opin Biotechnol.

[3] Davis K, Moon TS (2020). Tailoring microbes to upgrade lignin. Curr Opin Chem Biol.

[4] Beckham GT (2016). Opportunities and challenges in biological lignin valorization. Curr Opin Biotechnol.

[5] Patton AR, Gieseker L (1942). Seasonal changes in the lignin and cellulose content of some montana grasses. J Anim Sci.

[6] Vanholme R (2010). Lignin biosynthesis and structure. Plant Physiol.

[7] Mottiar Y (2016). Designer lignins: harnessing the plasticity of lignification. Curr Opin Biotechnol.

[8] Holder JW (2011). Comparative and functional genomics of *Rhodococcus opacus* pd630 for biofuels development. PLoS Genet.

[9] Alvarez H, Steinbüchel A (2002). Triacylglycerols in prokaryotic microorganisms. Appl Microbiol Biotechnol.

[10] Alvarez HM (1996). Formation of intracytoplasmic lipid inclusions by *Rhodococcus opacus* strain PD630. Arch Microbiol.

[11] Henson WR (2018). Multi-omic elucidation of aromatic catabolism in adaptively evolved *Rhodococcus opacus*. Metab Eng.

[12] Yoneda A (2016). Comparative transcriptomics elucidates adaptive phenol tolerance and utilization in lipid-accumulating *Rhodococcus opacus* PD630. Nucl Acid Res.

[13] Subramaniam R (2010). Microbial lipids from renewable resources: production and characterization. J Ind Microbiol Biotechnol.

[14] Hernandez MA (2013). The atf2 gene is involved in triacylglycerol biosynthesis and accumulation in the oleaginous *Rhodococcus opacus* PD630. Appl Microbiol Biotechnol.

[15] Alvarez AF (2008). Cloning and characterization of a gene involved in triacylglycerol biosynthesis and identification of additional homologous genes in the oleaginous bacterium *Rhodococcus opacus* PD630. Microbiology.

[16] Alvarez HM, Steinbüchel A, Alvarez HM (2019). Biology of triacylglycerol accumulation by *Rhodococcus*. Biology of *Rhodococcus*.

[17] Xie S (2019). Mechanism-guided design of highly efficient protein secretion and lipid conversion for biomanufacturing and biorefining. Adv Sci.

[18] Hernández MA, Alvarez HM (2019). Increasing lipid production using an NADP(+)-dependent malic enzyme from *Rhodococcus* jostii. Microbiology.

[19] Huang L (2016). Boosting fatty acid synthesis in *Rhodococcus opacus* PD630 by overexpression of autologous thioesterases. Biotech Lett.

[20] Villalba MS, Alvarez HM (2014). Identification of a novel ATP-binding cassette transporter involved in long-chain fatty acid import and its role in triacylglycerol accumulation in *Rhodococcus* jostii RHA1. Microbiology.

[21] Chen Y (2013). Integrated omics study delineates the dynamics of lipid droplets in *Rhodococcus* opacus PD630. Nucl Acid Res.

[22] Amara S (2016). Characterization of key triacylglycerol biosynthesis processes in *Rhodococci*. Sci Rep.

[23] Bai W (2022). Engineering diverse fatty acid compositions of phospholipids in *Escherichia coli*. Metab Eng.

[24] Jiang W (2017). Modular pathway engineering for the microbial production of branched-chain fatty alcohols. Biotechnol Biofuel.

[25] Breuer G (2013). Analysis of fatty acid content and composition in microalgae. J Vis Exp.

[26] Liu D, Zhang F (2018). Metabolic feedback circuits provide rapid control of metabolite dynamics. ACS Synth Biol.

[27] Bentley GJ (2016). Engineering *Escherichia coli* to produce branched-chain fatty acids in high percentages. Metab Eng.

[28] Bolger AM, Lohse M, Usadel B (2014). Trimmomatic: a flexible trimmer for Illumina sequence data. Bioinformatics.

[29] Li, H., Toolkit for processing sequences in FASTA/Q formats: lh3/seqtk. Github. 2022.

[30] Liao Y, Smyth GK, Shi W (2013). The subread aligner: fast, accurate and scalable read mapping by seed-and-vote. Nucl Acid Res.

[31] Wickham, H. and G. Grolemund. R for data science: import, tidy, transform, visualize, and model data. “O'Reilly Media, Inc”. 2016

[32] Love MI, Huber W, Anders S (2014). Moderated estimation of fold change and dispersion for RNA-seq data with DESeq2. Genome Biol.

[33] Kolde, R. and M.R. Kolde. Package ‘pheatmap’. R Package. 2018. 1.

[34] Kindt R, Kindt MR (2019). Package ‘biodiversity R’. Packag Commun Ecol Suitabil Anal.

[35] Dixon P (2003). VEGAN, a package of R functions for community ecology. J Veg Sci.

[36] Chen Y (2014). Integrated omics study delineates the dynamics of lipid droplets in Rhodococcus opacus PD630. Nucl Acid Res.

[37] Decanio MS, Landick R, Haft RJ (2013). The non-pathogenic *Escherichia coli* strain W secretes SslE via the virulence-associated type II secretion system beta. BMC Microbiol.

[38] Kaczanowska M, Rydén-Aulin M (2007). Ribosome biogenesis and the translation process in *Escherichia coli*. Microbiol Mol Biol Rev.

[39] Jain C, The E (2008). coli RhlE RNA helicase regulates the function of related RNA helicases during ribosome assembly. RNA.

[40] Díaz M (2005). The high-affinity phosphate-binding protein PstS is accumulated under high fructose concentrations and mutation of the corresponding gene affects differentiation in *Streptomyces* lividans. Microbiology.

[41] Nešvera, J., L. Rucká, and M. Pátek,. 2015. Chapter four—catabolism of phenol and its derivatives in bacteria: genes, their regulation, and use in the biodegradation of toxic pollutants, in advances in applied microbiology. In: S Sariaslani GM Gadd, (Eds.) Academic Press. Elsevier; Amsterdam. 107–160. 10.1016/bs.aambs.2015.06.002 26505690

[42] Li C (2016). Characterization of a protocatechuate catabolic gene cluster in *Rhodococcus* ruber OA1 involved in naphthalene degradation. Ann Microbiol.

[43] Whipp MJ, Camakaris H, Pittard AJ (1998). Cloning and analysis of the shiA gene, which encodes the shikimate transport system of Escherichia coli K-12. Gene.

[44] Straight JV, Ramkrishna D (1994). Modeling of bacterial growth under multiply-limiting conditions. experiments under carbon- or/and nitrogen-limiting conditions. Biotechnol Progress.

[45] Brigham, C.J., et al., Bacterial carbon storage to value added products. 2011.

[46] Peoples OP, Sinskey AJ. Poly-β-hydroxybutyrate (PHB) biosynthesis in *Alcaligenes eutrophus* H16: identification and characterization of the PHB polymerase gene (*phbC*) *. J Biol Chem. 1989;264(26):15298–303. 2670936

[47] Iuchi S, Lin EC (1987). The narL gene product activates the nitrate reductase operon and represses the fumarate reductase and trimethylamine N-oxide reductase operons in *Escherichia coli*. Proc Natl Acad Sci U S A.

[48] Mangalea MR, Borlee BR (2022). The NarX-NarL two-component system regulates biofilm formation, natural product biosynthesis, and host-associated survival in *Burkholderia pseudomallei*. Sci Rep.

[49] Ding Y (2012). Identification of the major functional proteins of prokaryotic lipid droplets. J Lipid Res.

[50] Zhang C (2017). Bacterial lipid droplets bind to DNA via an intermediary protein that enhances survival under stress. Nat Commun.

[51] Avidan O (2015). Enhanced acetyl-CoA production is associated with increased triglyceride accumulation in the green alga *Chlorella desiccata*. J Exp Bot.

[52] Zeng J, Spiro S (2013). Finely tuned regulation of the aromatic amine degradation pathway in *Escherichia coli*. J Bacteriol.

[53] Hooppaw AJ (2022). The phenylacetic acid catabolic pathway regulates antibiotic and oxidative stress responses in *Acinetobacter*. MBio.

[54] Chen R (2022). Engineering cofactor supply and recycling to drive phenolic acid biosynthesis in yeast. Nat Chem Biol.

