# Supplementary material for: Increased triacylglycerol production in Rhodococcus opacus by overexpressing transcriptional regulators
Source: Biotechnol Biofuels Bioprod. 2024 Jun 19;17:83. doi: 10.1186/s13068-024-02523-3 (PMC11186279; doi:10.1186/s13068-024-02523-3)
Supplement: Supplementary file 1 — Supplementary Table S1 Overexpression strains screened for nitrogen-independent lipid production in Rhodococcus opacus PD630. Table S2. Fatty acid standards for analysis of lipid samples extracted from Rhodococcus opacus PD60 cells. Figure S1. Potential native plasmid loss in multiple strains on growth in phenol and glucose. Figure S2. KEGG modules within the phenylalanine, tyrosine, and tryptophan biosynthesis and phenylalanine metabolism pathways with significant DE. Heatmap of KEGG module enrichment in each strain and carbon source. Each mutant was compared to WT grown using the same carbon source for differential expression in all the R. opacus’ annotated KEGG modules (rows). The color of each cell in the heatmap denotes the fold change versus WT, with white cells representing non-significant changes. Each column represents the DE of one condition, averaged from replicates and tested using GAGE in R. 33 Annotation bars on top of the heatmap denote the strain and carbon source in each column. Rows are clustered with the “complete” distance method using Minkoswki distance. Figure S4. KEGG reference pathway of phenylalanine metabolism, including the phenylacetic acid and phenylethylamine degradation modules. Network diagram representing gene/genes as rectangles and metabolites as nodes. Outlets to other reference pathways are denoted as ovals. [file 13068_2024_2523_MOESM1_ESM.docx]

## **Supplementary Data**

### **Tables**

#### **Supplementary Table 1.** **Overexpression strains screened for nitrogen-independent lipid production in *Rhodococcus opacus* PD630.**

| Overexpression Strain | Locus ID (ASM59954v1) | Locus ID (ASM2054278v1) | Gene Name (ASM59954v1) |
| --- | --- | --- | --- |
| PD630 + pWG001 | LPD06713 | K2Z90_RS31755 | yagI |
| PD630 + pWG002 | LPD02702 | K2Z90_RS12940 | liaR |
| PD630 + pWG005 | LPD01792 | K2Z90_RS08570 | degU |
| PD630 + pWG006 | LPD06436 | K2Z90_RS30445 | dosT |
| PD630 + pWG008 | LPD02128 | K2Z90_RS10170 | yobV |
| PD630 + pWG009 | LPD07239 | K2Z90_RS34290 | ybhD |
| PD630 + pWG012 | LPD06097 | K2Z90_RS28820 | BetI |
| PD630 + pWG013 | LPD06917 | K2Z90_RS32760 | paaX |
| PD630 + pWG014 | LPD03471 | K2Z90_RS16630 | yidP |
| PD630 + pWG015 | LPD07419 | K2Z90_RS35120 | ycbG |
| PD630 + pWG016 | LPD00835 | K2Z90_RS03985 | ttgR |
| PD630 + pWG018 | LPD07964 | K2Z90_RS37650 | yiaJ |
| PD630 + pWG019 | LPD06505 | K2Z90_RS30765 | kipR |
| PD630 + pWG020 | LPD03024 | K2Z90_RS14485 | Rv0472c-MT0489 |
| PD630 + pWG024 | LPD00827 | K2Z90_RS03945 | ttuE_C |
| PD630 + pWG025 | LPD01684 | K2Z90_RS08045 | yxaF |
| PD630 + pWG026 | LPD00567 | K2Z90_RS02690 | tcmR |
| PD630 + pWG027 | LPD07519 | K2Z90_RS35615 | slyA |
| PD630 + pWG028 | LPD02935 | K2Z90_RS14075 | Leu |
| PD630 + pWG029 | LPD03658 | K2Z90_RS17485 | HI_1364 |
| PD630 + pWG030 | LPD01130 | K2Z90_RS05395 | pro_trans |
| PD630 + pWG031 | LPD02085 | K2Z90_RS09960 | NarL |
| PD630 + pWG032 | LPD07217 | K2Z90_RS34190 | ttgR |
| PD630 + pWG033 | LPD05140 | K2Z90_RS24240 | Mb0601 |
| PD630 + pWG034 | LPD01132 | K2Z90_RS05405 | yagI |
| PD630 + pWG035 | LPD00075 | K2Z90_RS00365 | ribonuclease |
| PD630 + pWG036 | LPD06854 | K2Z90_RS32455 | famr |

Supplementary Table 2. Fatty acid standards for analysis of lipid samples extracted from *Rhodococcus opacus* PD60 cells.

| Short Notation | Long Notation | Linear Formula | Retention Time (min) |
| --- | --- | --- | --- |
| C12:0 | lauric/dodecanoic acid | CH_3_(CH_2_)_10_COOH | 6.23 |
| C14:0 | myristic/tetradecanoic acid | CH_3_(CH_2_)_12_COOH | 7.388 |
| C15:0 | pentadecanoic acid | CH_3_(CH_2_)_13_COOH | 7.929 |
| C16:1 | palmitoleic/hexadecenoic acid | CH_3_(CH_2_)_5_CH=CH(CH_2_)_7_COOH | 8.351 |
| C16:0 | palmitic/onadecanoic acid | CH_3_(CH_2_)_14_COOH | 8.443 |
| C16+Me | methyl palmitate/ hexadecanoate | CH_3_(CH_2_)_14_CO_2_CH_3_ | 8.624 |
| C17:1 | heptadecenoic acid | CH_3_(CH_2_)_5_CH=CH(CH_2_)_8_COOH | 8.804 |
| C17:0 | margaric/heptadecanoic acid | CH_3_(CH_2_)_15_COOH | 8.938 |
| C17+Me | methyl margarate/ heptadecanoate | CH_3_(CH_2_)_15_CO_2_CH_3_ | 9.12 |
| C18:1 | oleic/octadecenoic acid | CH_3_(CH_2_)_7_CH=CH(CH_2_)_7_COOH | 9.302 |
| C18:0 | stearic/octadecanoic acid | CH_3_(CH_2_)_16_COOH | 9.408 |
| C18+Me | methyl stearate/octadecanoate | CH_3_(CH_2_)_16_CO_2_CH_3_ | 9.57 |
| C19:1 | onadecanoic acid | CH_3_(CH_2_)_7_CH=CH(CH_2_)_8_COOH | 9.74 |
| C19:0 | nonadecanoic acid | CH_3_(CH_2_)_17_COOH | 9.862 |

### **Figures**


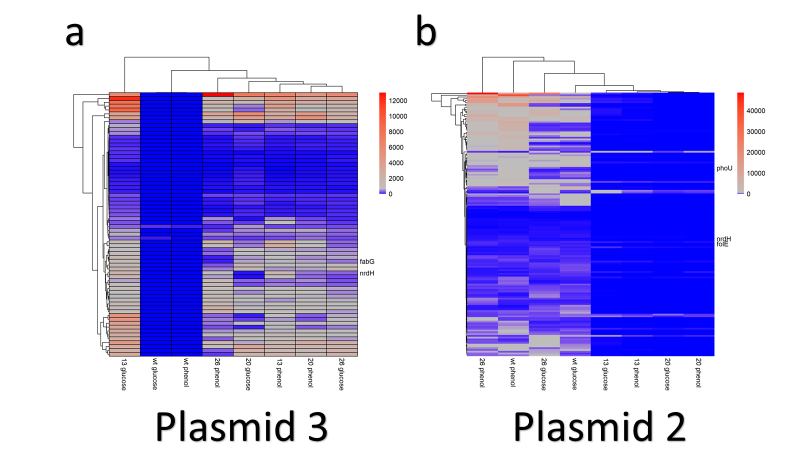


#### **Supplemental Figure 1.** Potential native plasmid loss in multiple strains on growth in phenol and glucose.

1. Raw transcript counts of all genes located on plasmid 3. Symbols of annotated genes appear along the right side of the heatmap. b. Raw transcript counts of all genes located on plasmid 2. Symbols of annotated genes appear along the right side of the heatmap.


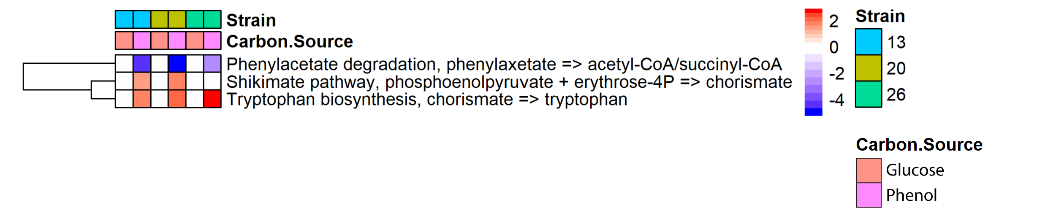


#### **Supplemental Figure 2.** KEGG modules within the phenylalanine, tyrosine, and tryptophan biosynthesis and phenylalanine metabolism pathways with significant DE.

Heatmap of KEGG module enrichment in each strain and carbon source. Each mutant was compared to WT grown using the same carbon source for differential expression in all the *R. opacus*’ annotated KEGG modules (rows). The color of each cell in the heatmap denotes the fold change versus WT, with white cells representing non-significant changes. Each column represents the DE of one condition, averaged from replicates and tested using GAGE in R. Annotation bars on top of the heatmap denote the strain and carbon source in each column. Rows are clustered with the “complete” distance method using Minkoswki distance.

**
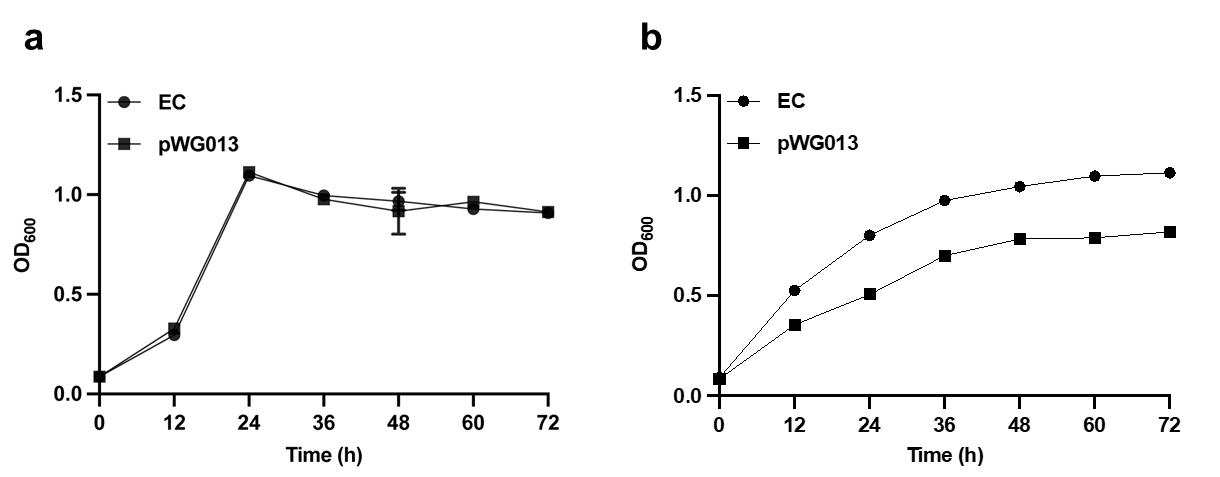
**

#### **Supplemental Figure 3.** Strain 13 grows at the same rate as WT in the glucose and nitrogen-replete condition, but with a lower OD in the nitrogen-limited condition. Growth curves of Strain 13 and WT grown in 2 g/L glucose and different nitrogen conditions. **a.** Nitrogen replete condition (1 g/L (NH_4_)_2_SO_4_)). **b.** Nitrogen limiting condition (0.05 g/L (NH_4_)_2_SO_4_). The initial OD was set to OD_600_=0.1, and cell density was measured every 12 hrs.


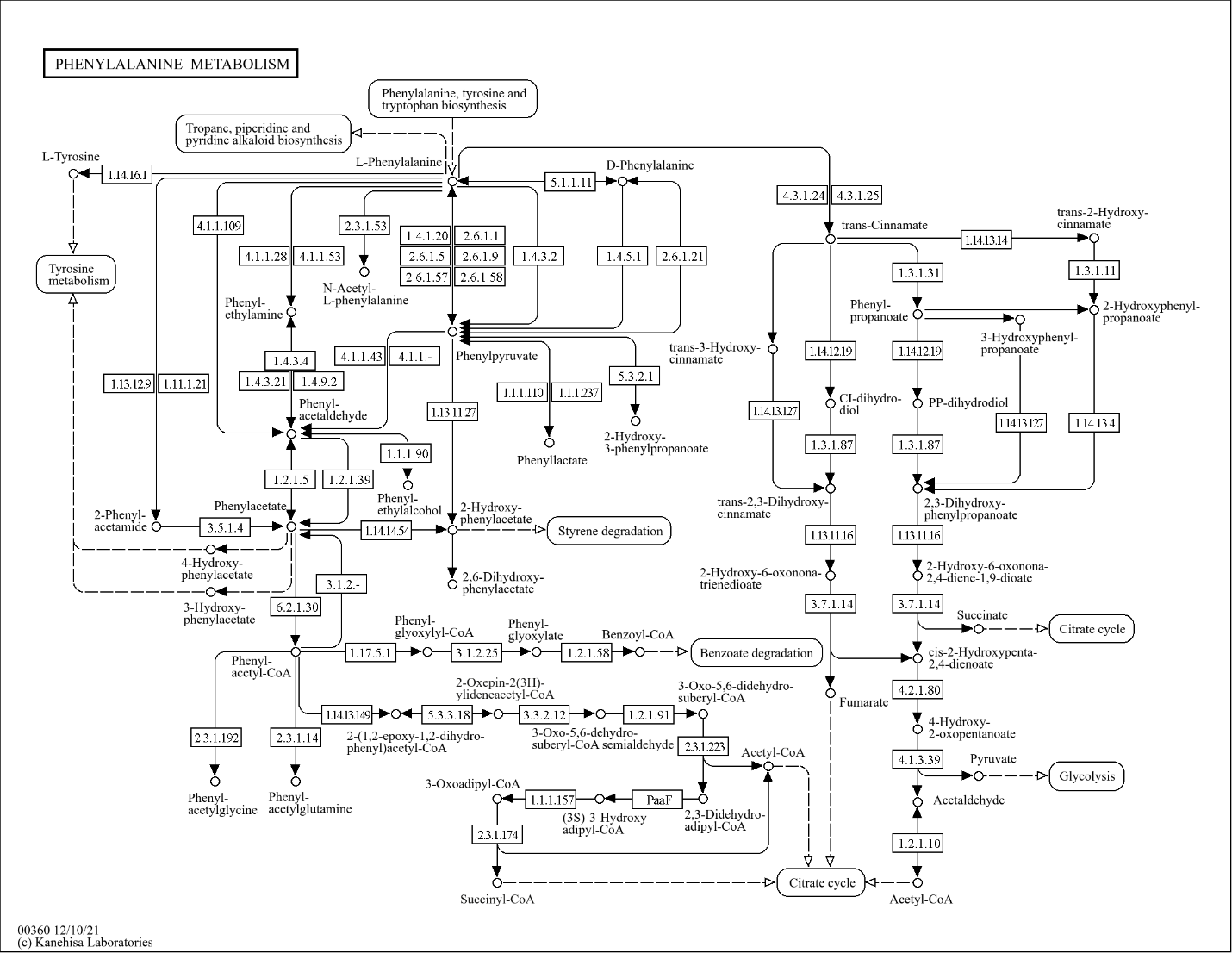


#### **Supplemental Figure 4.** KEGG reference pathway of phenylalanine metabolism, including the phenylacetic acid and phenylethylamine degradation modules. Network diagram representing gene/genes as rectangles and metabolites as nodes. Outlets to other reference pathways are denoted as ovals.
